# Establishing the history of safe use of the OsDIAT protein: preliminary characterization of the recombinant protein and *in silico* analyses

**DOI:** 10.1080/21645698.2026.2753265

**Published:** 2026-11-04

**Authors:** Jong-Chan Park, Sung-Dug Oh, Jae-Han Choi, Jung-Ho Park, Ji-Young Park, Hyoun-Min Park, Sang-Gu Lee, Seon-Woo Oh, An-Cheol Chang

**Affiliations:** a Department of Agricultural Biotechnology, National Institute of Agricultural Sciences, Rural Development Administration, Jeonju, Republic of Korea; b Bio-Evaluation Center, Korea Research Institute of Bioscience and Biotechnology, Cheongju, Republic of Korea; c Department of Applied Biological Engineering, KRIBB School of Biotechnology, Korea University of Science and Technology (UST), Daejeon, Republic of Korea

**Keywords:** Allergenicity, history of safe use, *in vitro* digestibility, OsDIAT, recombinant protein

## Abstract

The drought-induced branched-chain amino acid aminotransferase (OsDIAT) from *Oryza sativa* belongs to a protein family with a long history of safe use (HOSU). In this study, we conducted a preliminary characterization of the recombinant OsDIAT protein produced in *Escherichia coli*, together with *in silico* and literature-based analyses, to contribute to the weight of evidence supporting the HOSU of the OsDIAT protein. In simulated gastric fluid (SGF), the intact recombinant protein was no longer detectable within 1 min, whereas it remained detectable in simulated intestinal fluid (SIF) for 60 min in a separate time-course assay; sequential SGF–SIF digestion was not performed. When heated at 95 °C (the only temperature tested), the intact protein band was no longer detectable from 60 min onward; enzymatic activity was not measured. *In silico* analyses identified no significant sequence similarity to known proteinaceous toxins or allergens, and domain analysis was consistent with a pyridoxal phosphate-dependent aminotransferase. Two potential N-glycosylation sites were predicted, but because no signal peptide or transmembrane domain was predicted, the protein is not expected to enter the secretory pathway, and the sites are unlikely to be modified in the native protein. These preliminary data add to the weight of evidence supporting the HOSU of the OsDIAT protein. However, they relate only to the microbially produced recombinant protein, and further studies, including demonstration of equivalence with the plant-expressed protein, are required before these findings can be associated with any genetically modified plant expressing this protein.

## 1. Introduction

Rice (*Oryza sativa* L.) is a staple food, consumed by more than half of the global population. However, its production is continuously threatened by extreme climates, such as droughts, which have been exacerbated by global warming, posing a significant risk to global food security.^1,2^ Water deficiency impairs the photosynthetic apparatus of rice and induces oxidative stress, ultimately leading to a sharp decline in grain yield. Plants have evolved complex physiological defense mechanisms to adapt to extreme environments, including the accumulation of various secondary metabolites and amino acids that regulate intracellular osmotic pressure and detoxify reactive oxygen species.^3^ In particular, the interdependence of threonine and isoleucine metabolism under abiotic stress is crucial for maintaining cellular homeostasis.^4^ Furthermore, branched-chain amino acids (BCAAs), such as valine, leucine, and isoleucine, act as both precursors for protein synthesis and as potent osmoprotectants and stress-defense signaling molecules.^5^

Recent studies have revealed that the drought-induced BCAA aminotransferase (OsDIAT), a key enzyme in the BCAA metabolic pathway, plays a pivotal role in conferring drought tolerance in rice by regulating BCAA homeostasis. OsDIAT is a pyridoxal-5’-phosphate (PLP)-dependent enzyme. Overexpression of the *OsDIAT* gene in transgenic rice significantly increases the accumulation of BCAAs, remarkably improving survival rates and grain yield, including under severe water-deficit conditions.^6^ Information relevant to the safety of the OsDIAT protein is therefore of value for any future use of this protein in food or feed crops.

However, the introduction of novel proteins into the food and feed supply chain via genetic modification (GM) technology requires safety assessment, including evaluation of potential toxicity and allergenicity to humans and animals.^7^ International guidelines from the Codex Alimentarius Commission and European Food Safety Authority (EFSA) recommend a “weight-of-evidence” approach that synthesizes multiple lines of evidence rather than relying on a single experimental result.^8,9^ In practice, *in silico* analyses combined with *in vitro* digestion and thermal stability assays are commonly used as part of this approach, together with information on the history of the safe use (HOSU) of the source organism.^10–12^

In the safety assessment of newly expressed proteins in GM plants, utilizing a microbially-derived recombinant protein is standard practice, as obtaining sufficient quantities directly from plant tissues is challenging. However, to associate the safety data of a recombinant protein with a GM plant, additional work demonstrating biochemical equivalence to the plant-expressed protein is strictly required. Therefore, this study is not a comprehensive food safety assessment of a specific GM crop. Rather, it presents a preliminary characterization of the recombinant OsDIAT protein, together with *in silico* and literature-based analyses. In this study, we characterized the physicochemical properties of purified recombinant OsDIAT and analyzed its biological stability using *in vitro* digestion models with simulated gastric fluid (SGF) and simulated intestinal fluid (SIF) along with thermal treatment assays. Furthermore, *in silico* analyses were performed, including sequence homology comparisons with known toxins and allergens using up-to-date databases (e.g., AllergenOnline, ADFS), functional domain analysis, and the prediction of potential N-glycosylation sites.^11^ Ultimately, these preliminary studies are intended to contribute to the weight of evidence supporting the HOSU of the OsDIAT protein. Further data, including plant-expressed protein equivalence, will be required to associate these findings with the safety assessment of any future GM plant expressing this protein.

## 2. Materials and Methods

### 2.1. OsDIAT Recombinant Protein Expression and Purification

To generate the recombinant protein, the full-length coding sequence (CDS) of *OsDIAT* (Os05g0244700) from rice (*Oryza sativa* L. ssp. *japonica* “Nipponbare”) was amplified using specific primers (Table S1) and cloned into the pET26b (+) vector (Fig. S1). The resulting vector was transformed into *Escherichia coli* BL21 (DE3) cells. Laboratory-scale production of rOsDIAT was conducted to optimize culture conditions, including pH, dissolved oxygen (DO), and temperature, using a 7-L Jar bottom magnet drive fermenter (Lab Fermenter, CNS, Korea). The seed culture for fermentation was grown in 300 mL of Luria Bertani (LB) broth containing 50 µg/mL kanamycin with shaking at 180 rpm and 37 °C for 15 h. The seed culture was transferred into a 7-L fermenter containing 3 L of LB broth with 50 µg/mL kanamycin and incubated at 180 rpm and 37 °C until the bacterial cells reached the target optical density, with glucose fed during cultivation (fed-batch mode). Recombinant expression was induced with isopropyl β-D-1-thiogalactopyranoside (IPTG; 1 mM) at 0 h (OD_600_ ≈ 0.6). Then, protein expression was induced by incubation at 37 °C for 20 h, followed by further incubation at 25 °C for 4 h to enhance yield and optimize protein quality. For accurate monitoring of high cell-density, culture samples were appropriately diluted to maintain the absorbance readings within the linear range of the spectrophotometer (OD_600_ < 0.8), and the final OD_600_ was calculated by multiplying the reading by the dilution factor. Finally, the bacterial cells were harvested via centrifugation at 4,000 ×g for 30 min at 4 °C.

To purify rOsDIAT, harvested cells were resuspended in 50 mM sodium phosphate (pH 8.0) and subsequently lysed using a French press homogenizer (GEA Niro Soavi NS10014L2K; GEA, Italy) under cold conditions to extract rOsDIAT. The homogenate was centrifuged at 10,000 ×g for 30 min at 4 °C. The supernatant was removed and the insoluble pellet was collected. The pellet was resuspended in binding buffer (20 mM sodium phosphate, 500 mM NaCl, 8 M urea, 20 mM imidazole, 1 mM DTT, pH 8.0) and centrifuged again at 10,000 ×g for 30 min at 4 °C. The resulting supernatants were subjected to His-tagged affinity chromatography using a HisTrap HP column (Cytiva, 17,524,802) according to the manufacturer`s instructions. After passing the supernatant through a purification column, the column was washed with binding buffer five times to remove unbound proteins. Bound proteins were eluted, and urea was subsequently removed from the eluted protein by dialysis; the dialyzed protein was used for the assay. The molecular weight and sequence identification of purified rOsDIAT were determined using SDS-PAGE and western blotting. For this analysis, the proteins were separated on a 4–12% acrylamide gel and subsequently transferred to a PVDF membrane. The membrane was blocked with Tris-buffered saline (TBS) containing 5% skim milk for 1 h. Subsequently, the membrane was incubated sequentially with the primary antibody, a His-tag antibody (CST, 2365S), and the secondary antibody, an anti-rabbit IgG HRP-conjugated antibody (Abcam, ab6721). The final results were visualized using an enhanced chemiluminescence solution (Promega, W1001) according to the manufacturer`s instructions.

### 2.2. History of Safe Use of Oryza sativa and Derived Protein

A literature review on the HOSU of *O. sativa*, the source organism of the OsDIAT protein, was conducted following the principles outlined by Constable et al.^13^ A literature search was conducted using the PubMed database (https://pubmed.ncbi.nlm.nih.gov/) between February 19 and September 21, 2026, including articles published up to September 21, 2026. No start date restriction was applied to retrieve all available records. The keywords used for the search included a combination of the following terms, focusing on both the host species and the protein domain: (a) “*Oryza sativa*”; (b) “aminotransferase”; (c) “*Oryza sativa*” and “food safety”; (d) “*Oryza sativa*” and “risk assessment”; (e) “*Oryza sativa*” and “toxicity”; (f) “aminotransferase” and “safety”; (g) “aminotransferase” and “toxicity.” This review focused on reports concerning the long-term safe consumption of *O. sativa* (rice) and the general safety profile of the aminotransferase protein family to which OsDIAT belongs. Records were screened by title and abstract, and reports were considered relevant if they described the dietary consumption or safety of rice, or the safety or toxicity of aminotransferase proteins. In addition, background information on rice and on aminotransferases was obtained from published literature identified by the authors outside this search.

### 2.3. In Vitro Digestion Stability in Simulated Gastrointestinal Fluids

The *in vitro* pepsin digestion assay (SGF) and pancreatin digestion assay (SIF) were performed according to previously established standardized protocols^14–16^ with minor modifications. The *in vitro* digestibility of OsDIAT was evaluated using purified rOsDIAT derived from *E. coli* and incubation in SGF and SIF, performed as separate time-course assays (not sequentially).

#### 2.3.1. SGF Digestion

SGF was prepared by dissolving 3.2 mg/mL of pepsin (Sigma, P7012) in a 0.03 M sodium chloride solution, adjusted to pH 1.2. Aliquots of 200 µL of SGF were dispensed into 1.5 mL e-tubes and pre-incubated in a 37 °C water bath for 10 min. Digestion was initiated by adding 50 µg of rOsDIAT to each SGF aliquot, resulting in a pepsin-to-protein ratio of approximately 13:1 (w/w). The mixtures were incubated at 37 °C for periods of 0 s, 10 s, 30 s, 1 min, 10 min, 30 min, and 60 min. The reaction was immediately terminated by adding 75 µL of 0.2 M sodium carbonate to increase the pH. The 0 s sample was similarly treated by inactivating pepsin immediately after mixing. The resulting samples were mixed with 5×SDS-PAGE loading dye, heated at 100 °C for 10 min, and analyzed using SDS-PAGE. For precise confirmation, parallel SDS-PAGE was performed under identical conditions, followed by the transfer of proteins to a PVDF membrane and subsequent western blot analysis using an alkaline phosphatase (AP)-conjugated anti-His-tag antibody. Band detection was achieved by incubating the membrane with BCIP®/NBT (5-bromo-4-chloro-3-indolyl phosphate/nitro-blue tetrazolium chloride; Sigma, B1911) solution for chromogenic detection.

#### 2.3.2. SIF Digestion

SIF was prepared by dissolving 10 mg/mL pancreatin (Sigma, P7545) in a 0.05 M potassium phosphate monobasic solution, adjusted to pH 7.5. Aliquots of 64 µL of SIF were dispensed into 1.5 mL e-tubes and pre-incubated at 37 °C for 10 min. Digestion was performed by adding 50 µg of rOsDIAT to the SIF aliquots, whilst maintaining a pancreatin-to-protein ratio of approximately 13:1 (w/w). Then, the mixtures were incubated at 37 °C for 0 s, 10 s, 30 s, 1 min, 10 min, 30 min, and 60 min. The samples were processed and analyzed using SDS-PAGE and western blotting following procedures identical to those used for SGF digestion.

### 2.4. Thermal Stability of OsDIAT

The thermal stability of OsDIAT was evaluated using purified rOsDIAT derived from *E. coli* according to previously described methods^17^ with minor modifications. For the assay, 0.3 µg of rOsDIAT was prepared in 1.5 mL e-tubes and subjected to heat treatment at 95 °C for periods of 0, 10, 20, 30, 60, 90, 120, and 150 min. After the specified incubation time, the reaction was immediately terminated by rapidly cooling the tubes on ice. Then, the heat-treated samples were prepared for analysis by adding 5×SDS-PAGE loading dye and heated at 95 °C for 10 min. The resulting samples were processed and analyzed by SDS-PAGE and western blotting using the same procedures described for SGF digestion analysis.

### 2.5. Bioinformatics Analysis

#### 2.5.1. Similarity Search in NCBI Non-Redundant Database

The full-length amino acid sequence of OsDIAT (GenBank accession No. BAS92991.1) was retrieved from the NCBI protein database and used as the query sequence for all *in silico* safety assessments. The sequence was initially screened against the NCBI for Biotechnology Information non-redundant (NR) protein database (http://www.ncbi.nlm.nih.gov/BLAST/). This comparison was performed using the BLASTP algorithm (version 2.17.0+) with the BLOSUM62 scoring matrix. A nonrestrictive search was performed to capture all homologous proteins, regardless of annotation. As there are no universally accepted criteria for toxic protein screening,^18^ the significance of the sequence matches was evaluated. Matches were considered relevant if they met a stringent E-value < 0.01 and demonstrated ≥ 50% sequence identity over the full length of the protein, or ≥35% identity over a significant contiguous stretch of 80 amino acids or more.^8^ The corresponding protein identifiers (IDs) of the relevant hits were cross-referenced in the UniProt database (https://www.uniprot.org/) and further investigated using the NCBI database for adverse effects. All analyses were completed on February 28, 2026.

#### 2.5.2. Similarity Search Using Allergen Databases and Allergen Motifs

The allergenic potential of the OsDIAT amino acid sequence was evaluated by comparing sequences with known allergenic proteins, following the general recommendations of the Codex Alimentarius.^8,19^ The OsDIAT sequence was analyzed using the BLASTp algorithm against the AllergenOnline version 24 database (http://www.allergenonline.org/), which is a widely used, curated resource for allergenic proteins. Supplementary analyses were also conducted using the Allergen Database for Food Safety (ADFS) (https://allergen.nihs.go.jp/) and the Allermatch™ tool (http://www.allermatch.org/) to broaden the scope of the search. Three distinct search criteria were used in the analysis:

1. Full-Length Sequence Similarity: The complete sequence was subjected to a BLASTp search. Matches were considered potentially relevant if the alignment resulted in at least 50% sequence identity with the full-length protein.^20^ The E-value cutoff was set at 0.01.
2. 80 Amino Acid Window Similarity: To identify potential structural motifs that may contribute to cross-reactivity, the OsDIAT sequence was evaluated for any 80 contiguous amino acid stretch that displayed ≥ 35% sequence identity with a known allergen.^21^
3. Contiguous Motif Search (Epitope Screening): The sequence was screened for short linear IgE-binding epitopes. This search specifically targeted the presence of any six, seven, or eight contiguous amino acid sequences that showed 100% identity to the corresponding segments of any known allergenic protein.^8^

All search results satisfying these three criteria were reviewed against source allergen information to determine their biological relevance. All analyses were completed on February 28, 2026.

#### 2.5.3. Functional Domain Analysis and Mode of Action (MOA) Prediction

The potential functional domains and predicted biochemical activity of the OsDIAT protein were analyzed to assess its potential mode of action (MOA) and specificity, following recommendations for protein safety assessment.^10^ The primary amino acid sequence of OsDIAT was subjected to domain and family analyses using the Pfam (http://pfam.xfam.org/) and InterPro (http://www.ebi.ac.uk/interpro/) databases. These analyses were conducted to identify known functional domains, binding sites, and structural homologies that could indicate uncharacterized toxicity or anti-nutritional activities. Subsequently, a targeted literature search was performed to gather information regarding the known or predicted MOA and the specificity of the OsDIAT protein and its closest functional homologs identified in Section 2.5.1. The PubMed database (http://www.ncbi.nlm.nih.gov/pubmed/) was accessed using specific combinations of keywords tailored to the protein: (a) “OsDIAT” and “mode of action”; (b) “OsDIAT” and “specificity”; (c) “OsDIAT” and “inhibitor” or “toxicity”; (d) “mode of action” and “D-amino-acid transaminase.” All searches and data retrievals were performed on February 28, 2026, to use the most current literature available at that time.

#### 2.5.4. N-Glycosylation Site Prediction

The full-length amino acid sequence of OsDIAT was analyzed to predict the presence of potential N-glycosylation sites. This analysis provides information on potential N-linked post-translational modifications. Predictions were made using the NetNGlyc 1.0 Server program (https://services.healthtech.dtu.dk/services/NetNGlyc-1.0/). N-Glycosylation sites were defined using the standard sequence motif Asn-X-Ser/Thr (where X represents any amino acid except Pro). The search was conducted on February 28, 2026. The presence of an N-terminal signal peptide was predicted using SignalP 6.0 (https://dtu.biolib.com/SignalP-6)^22^ and the presence of transmembrane domains was predicted using DeepTMHMM (https://dtu.biolib.com/DeepTMHMM),^23^ using the full-length OsDIAT sequence as input. The analysis was performed on September 20, 2026.

## 3. Results and Discussion

### 3.1. Expression, Purification, and Identification of the Recombinant OsDIAT Protein

To obtain sufficient quantities of the OsDIAT protein for the characterization studies, a microbial surrogate expression system was employed. This is a standard and internationally recognized approach for the safety assessment of GM crops because extracting adequate amounts of target proteins directly from plant tissues is often not practically feasible.^7,10^ The full-length coding sequence of *OsDIAT* (Os05g0244700) was successfully cloned into a pET26b (+) vector and expressed as a recombinant protein (rOsDIAT) in *Escherichia coli* BL21 (DE3) cells. Based on the primary amino acid sequence, the theoretical molecular weight of rOsDIAT, including its C-terminal His-tag, was approximately 37.12 kDa.

High-yield production of rOsDIAT was achieved using fed-batch cultivation in a 7-L fermenter. The *E. coli* culture reached a high final optical density (OD_600_) of 114 (Fig. 1A). Recombinant protein expression was induced with IPTG at an OD_600_ of 0.6. To optimize the protein yield and minimize premature degradation, the production phase was conducted under the established optimal fed-batch condition. Figure 1B illustrates the successful expression profile of the recombinant protein under this optimized condition.

**Figure 1.**
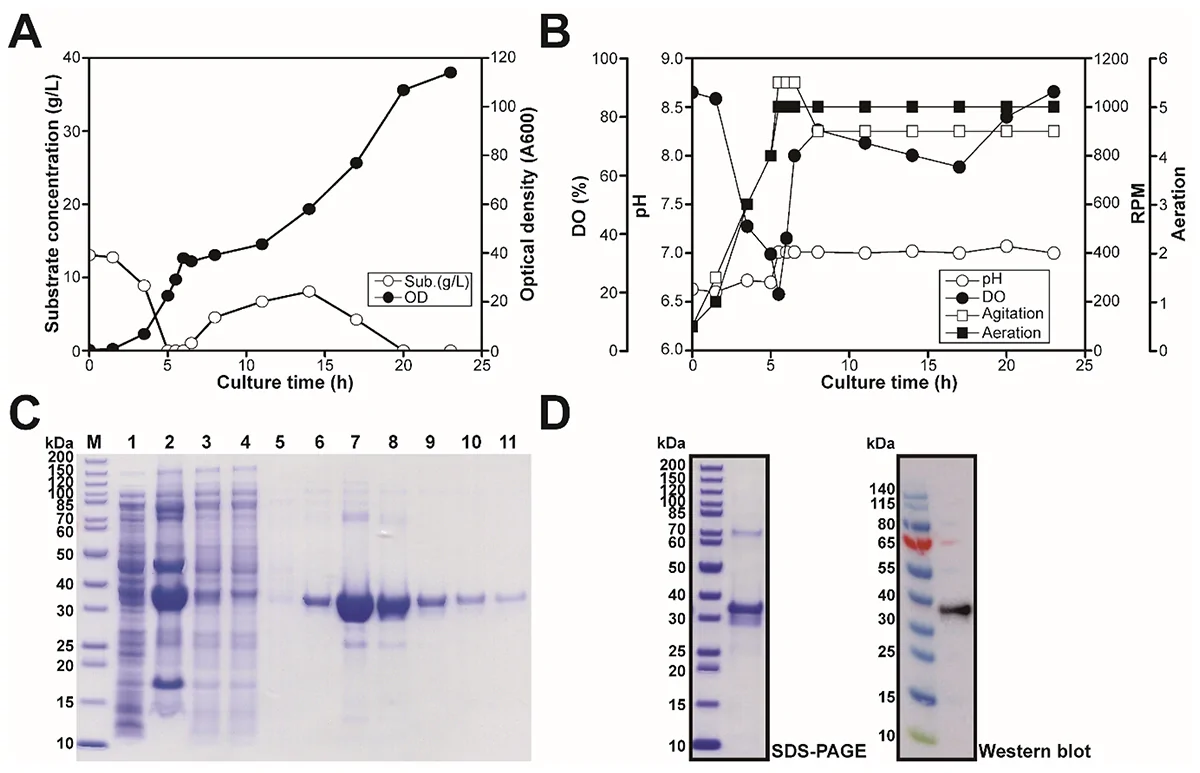
Production, purification, and identification of recombinant OsDIAT protein. (A) fed-batch cultivation profile (OD_600_ and glucose consumption). (B) fermentation parameters. Monitoring of dissolved oxygen (DO), pH, agitation speed (RPM), and aeration. IPTG induction was performed at 0 h, followed by a two-stage temperature shift for protein production. (C) SDS-PAGE (4–12%) analysis of rOsdiat purification fractions. M: marker; 1: whole cell lysate; 2: Supernatant; 3: pellet (insoluble fraction); 4: flow-through; 5: washing fraction; 6–11: elution fractions. (D) identity confirmation of purified rOsdiat protein. SDS-PAGE (left panel) and western blot (right panel) confirmed the protein identity at the predicted size of 37.12 kDa.

Fractionation analysis revealed that rOsDIAT was primarily expressed in the insoluble inclusion body fraction (Fig. 1C). Subsequently, the protein was solubilized and purified using His-tag affinity chromatography under denaturing conditions. SDS-PAGE of the eluted fractions revealed a single predominant protein band corresponding to an expected molecular mass of approximately 37 kDa (Fig. 1D). The identity of the purified protein was further confirmed by western blotting using an anti-His-tag antibody, which yielded a distinct signal at the predicted molecular weight (Fig. 1D). A highly pure protein preparation (>95%) is desirable for *in vitro* digestion and thermal stability assays so that the results reflect the properties of the target protein rather than those of microbial impurities.^11^

### 3.2. *HOSU of* Oryza Sativa *and the Aminotransferase Protein Family*

The evaluation of the HOSU of the source organism is one component of the weight of evidence for a novel protein. *Oryza sativa* (rice) has been cultivated and consumed worldwide as a primary staple food for thousands of years, with a long documented history of human consumption. Moreover, rice is widely recognized for its high nutritional value and beneficial health properties.^24,25^

The PubMed search (Section 2.2) retrieved 57,696 records for “*Oryza sativa*” (a) and 115,451 records for “aminotransferase” (b); because of their size, these two general queries were not screened individually. The combined queries (c)–(g) retrieved 3, 130, 1,187, 5,129 and 8,023 records, respectively. The three records retrieved by query (c) were reviewed in full: they concerned acrylamide formation in a black rice-enriched food, the safety evaluation of a partially hydrolyzed rice and barley protein as a novel food, and cadmium accumulation in rice grown in Chinese soils, and none reported adverse effects of rice proteins or addressed aminotransferases. For queries (d)–(g), the records were sorted by best match, and the first 50 records of each query were screened by title and abstract. None of the records screened was considered relevant; the aminotransferase queries in particular retrieved records that included studies using serum aminotransferase activities (ALT and AST) as biomarkers of liver injury, rather than studies on the safety of dietary aminotransferase proteins. No report of toxicity or adverse effects attributable to rice aminotransferases or to the OsDIAT protein was therefore identified among the records screened. Accordingly, the HOSU of the OsDIAT protein is supported by the long history of rice consumption and the ubiquitous dietary exposure to aminotransferases described below, together with published background literature, rather than by protein-specific safety studies.

Unlike other staple cereals (e.g., wheat, barley, and rye), rice is naturally gluten-free, and rice-based foods are commonly used in gluten-free diets, although, as with other foods, allergy to certain rice proteins has been reported.^26^ While some public health concerns have been raised regarding arsenic accumulation associated with rice consumption, this contamination is largely attributed to environmental factors (e.g., soil and water conditions) rather than to an intrinsic toxic property of the crop.^27^

Furthermore, the intrinsic safety of the aminotransferase (transaminase) protein family, to which OsDIAT belongs, provides additional supporting information. Aminotransferases are ubiquitous metabolic enzymes found across all domains of life, including plants, animals, and microorganisms. Consequently, humans and animals are continuously exposed to diverse, functionally homologous aminotransferases in substantial quantities through the standard dietary intake of conventional whole foods. In mammals, these enzymes are endogenously expressed and play critical physiological roles in amino acid metabolism. The long-standing and ubiquitous dietary exposure to this protein family, together with the long history of consumption of the rice source crop, contributes to the weight of evidence supporting the HOSU of the OsDIAT protein. This information relates to the source organism and the protein family and does not replace protein-specific data.

### 3.3. Stability of OsDIAT Protein in SGFs

#### 3.3.1. SGF Digestion

The susceptibility of a novel protein to proteolytic degradation in the human gastrointestinal tract is a key parameter in assessing its potential allergenicity and toxicity.^7,8^ Rapid degradation by pepsin in SGF is generally regarded as one line of evidence that a protein is less likely to reach the intestinal mucosa in an intact form.^28,29^ However, digestibility alone is not a reliable predictor of allergenicity or toxicity.^30^

The *in vitro* digestibility of the purified rOsDIAT protein was evaluated by incubation in SGF (pH 1.2) containing pepsin. As depicted in Fig. 2A, SDS-PAGE revealed a distinct band corresponding to the full-length rOsDIAT protein at the initial time point (0 s). Following exposure to SGF, this intact protein band was rapidly hydrolyzed and became visually undetectable within the first minute of incubation, and it remained undetectable throughout the 60-min assay period (Fig. 2A). To validate these findings with enhanced sensitivity, western blot analysis using an anti-His-tag antibody was performed in parallel. The immunoblot confirmed that, while trace amounts of the full-length protein were faintly visible at 30 s, the specific signal for rOsDIAT was degraded below the limit of detection by 1 min (Fig. 2B). These results indicate that the intact rOsDIAT protein was rapidly degraded by pepsin under the conditions tested.

**Figure 2.**
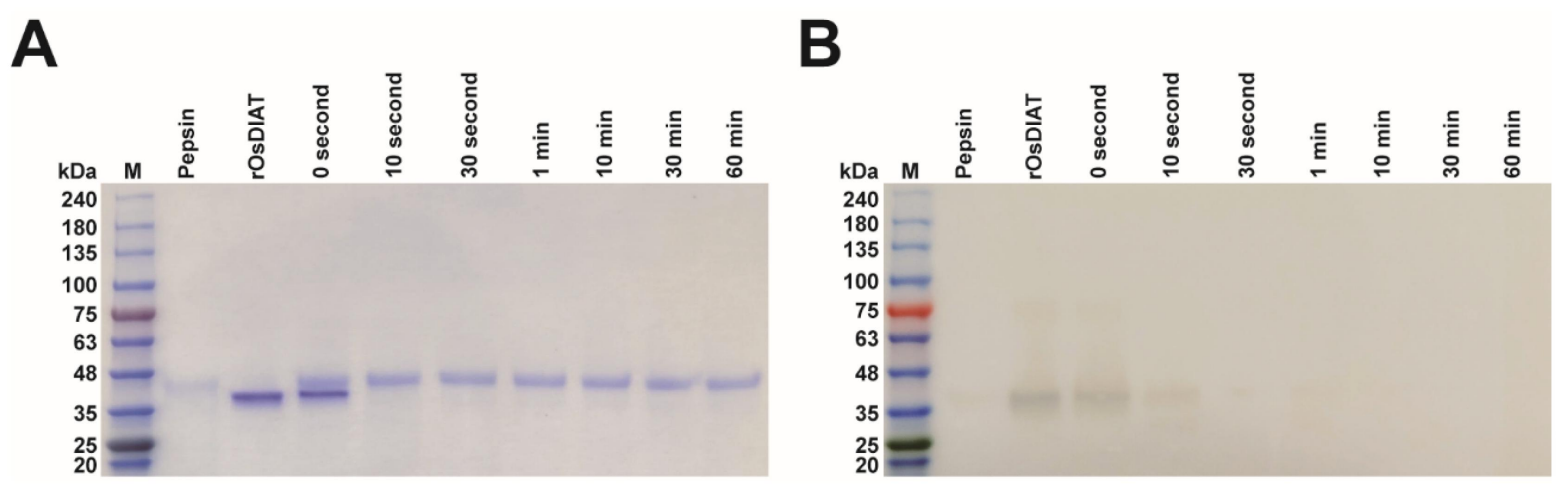
*In vitro* digestion stability of rOsdiat in simulated gastric fluid (SGF). The stability of purified rOsdiat protein (~37 kDa) was evaluated after incubation in SGF (pH 1.2) containing pepsin at a pepsin-to-protein ratio of approximately 13:1 (w/w). Incubation times ranged from 0 s to 60 min, as indicated above the lanes. (A) SDS-PAGE (4–12%) analysis showing the overall degradation profile of the full-length rOsdiat protein over time. (B) western blot analysis using anti-His-tag antibody for high-sensitivity chromogenic detection, showing that the intact rOsdiat signal was no longer detectable by the 1 min time point.

The rapid degradation observed is consistent with the behavior of proteins that are readily digested in SGF, which has been considered as one factor in the assessment of potential allergenicity.^21^ Because the intact protein was no longer detectable within 1 min, it is considered unlikely that intact rOsDIAT would persist long enough in the stomach to reach the small intestine under the conditions tested. However, digestibility is only one element of the weight of evidence, and the fate of peptide fragments was not evaluated: the anti-His-tag antibody detects only fragments that retain the C-terminal tag, and small fragments may not be detected by the gels used. In addition, because the protein was purified under denaturing conditions and dialyzed without verification of its native conformation, its susceptibility to pepsin may differ from that of the native or plant-expressed protein. These findings therefore provide only limited *in vitro* evidence that contributes to the weight of evidence supporting the HOSU of the OsDIAT protein.^11^

#### 3.3.2. SIF Digestion

Following evaluation using the gastric model, the susceptibility of rOsDIAT to intestinal proteases was assessed using SIF containing pancreatin (pH 7.5 for up to 60 min). As depicted in Fig. 3A, SDS-PAGE revealed that the full-length rOsDIAT protein (~37 kDa) remained intact and was clearly visible at all time points up to 60 min. The intensity of the protein band did not significantly decrease throughout the incubation period, indicating resistance to pancreatin digestion under the conditions tested. This pronounced stability in SIF was corroborated by western blot analysis using an anti-His-tag antibody, wherein the specific immunoreactive signal for rOsDIAT persisted without noticeable degradation for 60 min (Fig. 3B).

**Figure 3.**
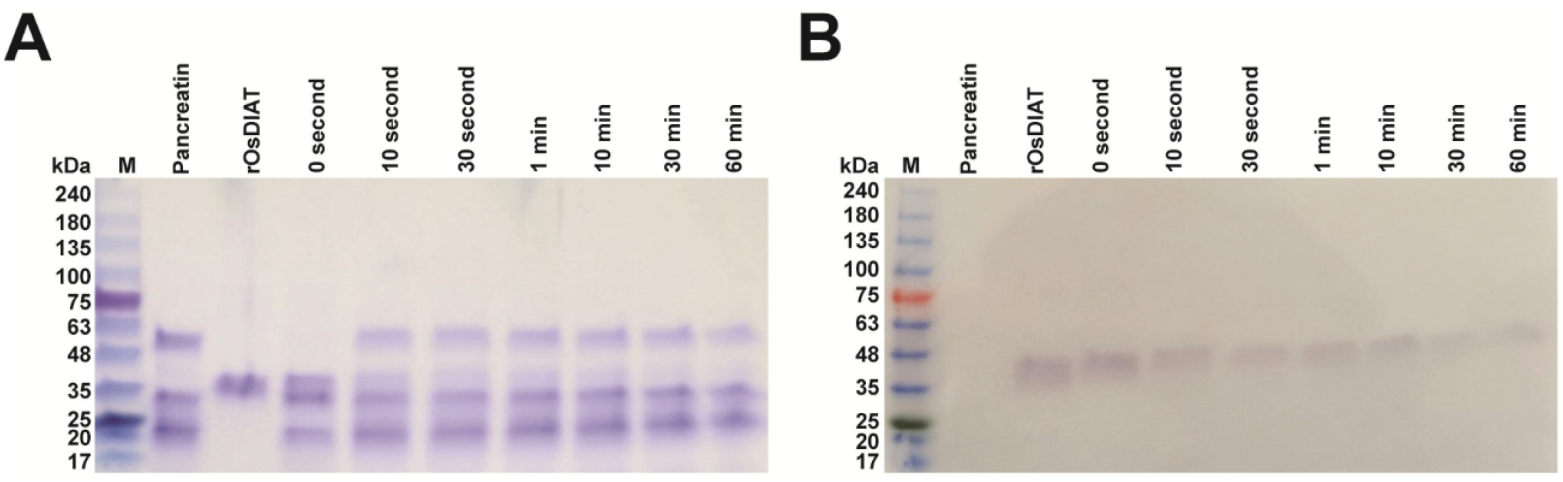
*In vitro* digestion stability of rOsdiat in simulated intestinal fluid (SIF). The purified rOsdiat protein (~37 kDa) was incubated in SIF (pH 7.5) containing pancreatin at a pancreatin-to-protein ratio of approximately 13:1 (w/w) for periods ranging from 0 s to 60 min. (A) SDS-PAGE (4–12%) analysis showing the overall stability profile of the full-length rOsdiat protein over time in the SIF environment. (B) western blot analysis using anti-His-tag antibody. The intact rOsdiat signal remained detectable throughout the 60 min incubation period.

The resistance of intact rOsDIAT to pancreatin observed in this separate time-course assay should be interpreted in the context of the physiological sequence of digestion. In the physiological continuum of mammalian digestion, ingested dietary proteins are sequentially subjected to the highly acidic, pepsin-rich environment of the stomach prior to entering the duodenum.^30^ As described in Section 3.3.1, the intact rOsDIAT protein was no longer detectable after 1 min in SGF. It is therefore considered unlikely that the intact protein would reach the intestinal lumen in vivo, although this was not tested in a sequential digestion. In this context, the *in vitro* SIF stability of the intact protein may not represent a realistic exposure scenario for dietary exposure and systemic sensitization.^11^ Furthermore, sequential digestion in SIF following a digestion in SGF is typically used to assess the susceptibility of low molecular weight SGF fragments. Such a sequential digestion was not performed in this study. It would further support the HOSU of the protein and is a recommended study to contribute to the safety assessment of a GM plant expressing this protein.

### 3.4. Thermal Stability of the OsDIAT Protein

Evaluating the thermal stability of a novel protein is one component of the weight-of-evidence approach, as it is indicative of the physical and structural changes that the protein may undergo during standard food processing and domestic preparation (e.g., cooking or boiling).^31^ Heat treatment can induce protein denaturation, which may lead to the loss of functional activity and to changes in conformational epitopes.

The thermal stability of the purified rOsDIAT protein (~37 kDa) was evaluated by incubating the protein at 95 °C for up to 150 min and monitoring the intact protein band (Fig. 4). As depicted in Fig. 4A, SDS-PAGE analysis showed a time-dependent reduction in the intensity of the intact rOsDIAT band. The band was detectable at the initial time point (0 s), decreased progressively during the first 20 min of heat exposure, and was barely detectable at 30 min. To corroborate these findings with higher sensitivity, western blotting was performed using an anti-His-tag antibody (Fig. 4B). The specific immunoreactive signal for the full-length protein was still faintly detectable at 30 min; however, from 60 min of heating onward (up to 150 min), the band corresponding to the intact rOsDIAT protein was no longer detectable by either SDS-PAGE or western blotting. This progressive loss of the intact protein band indicates that prolonged heating at 95 °C reduced the amount of intact protein detectable by these methods; the underlying process (e.g., denaturation, aggregation, or fragmentation) was not examined. Two limitations should be noted. First, only a single temperature (95 °C) was tested, whereas heat stability is typically evaluated over a range of temperatures (e.g., 25, 50, 75, and 95 °C); the present data therefore do not describe the temperature dependence of the stability of the protein and should be regarded as preliminary. Second, only the presence of the intact protein band was monitored, and enzymatic activity was not measured. Because the presence of a band does not indicate retained activity, and the loss of a band is not a direct measure of the loss of activity, demonstrating the loss of enzymatic activity over time under heat conditions would be a valuable complementary characterization for this protein.

**Figure 4.**
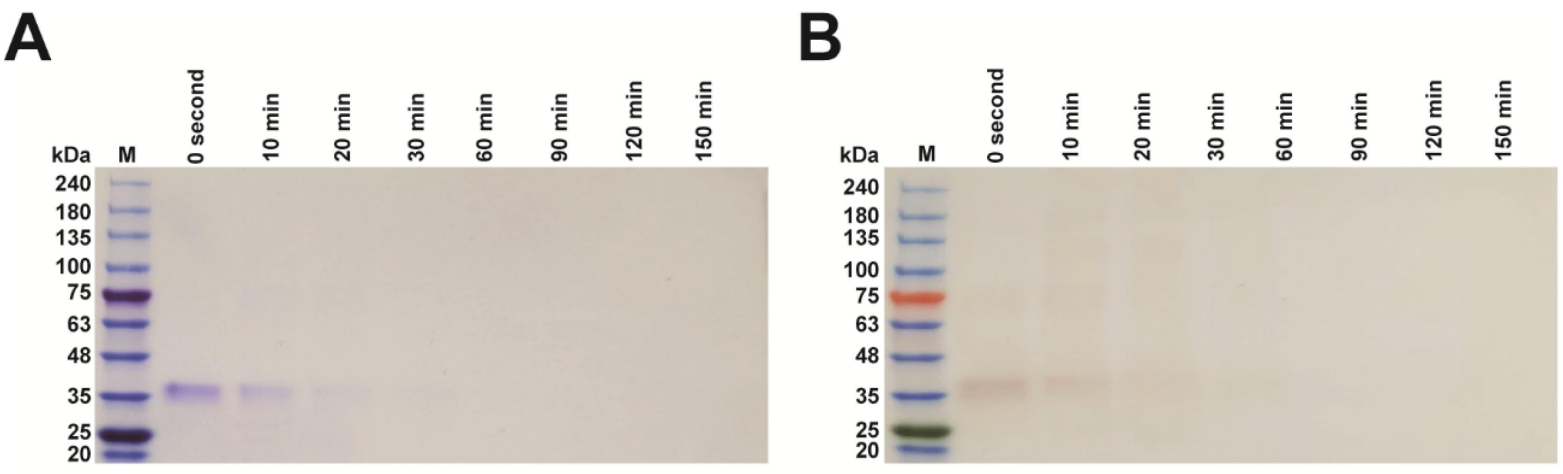
Thermal stability of the rOsdiat protein. The purified rOsdiat protein (~37 kDa) was exposed to 95 °C for periods ranging from 0 s to 150 min. (A) SDS-PAGE (4–12%) analysis. Shows the stability profile of the full-length rOsdiat protein over time under heat stress. (B) western blot analysis. Anti-His-tag antibody was used to detect the intact rOsdiat protein; the signal was no longer detectable from the 60 min time point onward.

The observed decrease of the intact protein band indicates that OsDIAT is susceptible to prolonged heating at 95 °C under the conditions tested. Foods are commonly heated during processing and domestic preparation; however, the temperatures, durations, and food matrices involved vary and were not simulated in this study, and the extent to which typical food processing would affect this protein therefore cannot be concluded from these data. Together with the results obtained in SGF, these findings provide preliminary information on the stability of the recombinant protein and add to the weight of evidence, but they do not by themselves demonstrate the absence of exposure to intact or functional OsDIAT.^11^

### 3.5. In Silico Safety Analyses

#### 3.5.1. Sequence Homology Analysis of Potential Toxicity (NCBI NR Database)

One step in the weight-of-evidence safety assessment of a novel protein is an *in silico* investigation of its amino acid sequence to identify any potential similarities with known proteinaceous toxins or anti-nutritional factors.^7,8^ To assess the potential toxicity of OsDIAT, a sequence homology search was conducted against the National Center for Biotechnology Information (NCBI) NR protein database using the BLASTP algorithm.

The search results, summarized in Table 1, showed that OsDIAT shares high sequence identity with proteins from a range of plant species, including agricultural crops that are widely consumed, particularly species within the Poaceae (grass) family. The most significant alignment identified was an orthologous aminotransferase from *O. sativa* (accession no. XP_015638016.1), followed by putative aminotransferases from the same species. High sequence homology with proteins across major global food crops, including sorghum (*Sorghum bicolor*), proso millet (*Panicum miliaceum*), barley (*Hordeum vulgare*), wheat (*Triticum aestivum*), and bananas (*Musa acuminata*), was also observed, which is consistent with the presence of this protein family in commonly consumed plant foods.

**Table 1.** Protein matches for the full-length amino acid sequences of OsDIAT in the NCBI non-redundant (nr) protein sequences database^a^.

Protein matched; ID; [*Species*]	Protein name^b^	Adverse effects reported^c^
D–amino–acid transaminase; XP_015638016.1; [*Oryza sativa*]	NA^d^	NP^e^
Putative aminotransferase; AAV43971.1; [*Oryza sativa*]	Aminotransferase	–^f^
D–amino–acid transaminase; XP_052155169.1; [*Oryza glaberrima*]	Uncharacterized protein	–
Hypothetical protein OsI_19143; EEC78836.1; [*Oryza sativa*]	Uncharacterized protein	–
Hypothetical protein E2562_028408; KAF0926905.1; [*Oryza meyeriana*]	Uncharacterized protein	–
Hypothetical protein E2562_028408; KAF0926908.1; [*Oryza meyeriana*]	Uncharacterized protein	–
D–amino–acid transaminase; XP_006655133.1; [*Oryza brachyantha*]	NA	NP
Hypothetical protein E2562_028408; KAF0926918.1; [*Oryza meyeriana*]	Uncharacterized protein	–
Hypothetical protein GUJ93_ZPchr0005g14839; KAG8067582.1; [*Zizania palustris*]	Uncharacterized protein	–
Hypothetical protein E2562_028408; KAF0926900.1; [*Oryza meyeriana*]	Uncharacterized protein	–
Hypothetical protein E2562_028408; KAF0926901.1; [*Oryza meyeriana*]	Uncharacterized protein	–
Hypothetical protein E2562_028408; KAF0926902.1; [*Oryza meyeriana*]	Uncharacterized protein	–
Hypothetical protein E2562_028408; KAF0926903.1; [*Oryza meyeriana*]	Uncharacterized protein	–
Hypothetical protein E2562_028408; KAF0926913.1; [*Oryza meyeriana*]	Uncharacterized protein	–
Hypothetical protein E2562_028408; KAF0926914.1; [*Oryza meyeriana*]	Uncharacterized protein	–
Unnamed protein product; CAL4903566.1; [*Urochloa decumbens*]	Uncharacterized protein	–
Unnamed protein product; CAL4910982.1; [*Urochloa decumbens*]	Uncharacterized protein	–
D–amino–acid transaminase; XP_062180851.1; [*Phragmites australis*]	NA	NP
Unnamed protein product; CAL4903567.1; [*Urochloa decumbens*]	Uncharacterized protein	–
Unnamed protein product; CAL5097680.1; [*Urochloa decumbens*]	Uncharacterized protein	–
D–amino–acid transaminase; XP_066368524.1; [*Miscanthus floridulus*]	NA	NP
Unnamed protein product; CAD6334235.1; [*Miscanthus lutarioriparius*]	Uncharacterized protein	–
Unnamed protein product; CAL4896362.1; [*Urochloa decumbens*]	Uncharacterized protein	–
Unnamed protein product; CAL5097681.1; [*Urochloa decumbens*]	Uncharacterized protein	–
D–amino–acid transaminase; XP_002440849.1; [*Sorghum bicolor*]	NA	NP
Hypothetical protein EJB05_34668; TVU18562.1; [*Eragrostis curvula*]	Uncharacterized protein	–
Hypothetical protein GQ55_3G066900; PUZ63419.1; [*Panicum hallii*]	Uncharacterized protein	–
Unnamed protein product; CAL4896363.1; [*Urochloa decumbens*]	Uncharacterized protein	–
Hypothetical protein BDA96_09G093800; KAG0517492.1; [*Sorghum bicolor*]	Uncharacterized protein	–
Hypothetical protein BS78_02G083400; KAJ1288355.1; [*Paspalum vaginatum*]	NA	NP
Protein matched; ID; [*Species*]	Protein name	Adverse effects reported
Hypothetical protein C2845_PM06G10850; RLM99778.1; [*Panicum miliaceum*]	D-amino-acid transaminase	–
D–amino–acid transaminase; XP_025807946.1; [*Panicum hallii*]	NA	NP
D–amino–acid transaminase; XP_039833461.1; [*Panicum virgatum*]	NA	NP
Hypothetical protein QOZ80_5AG0400620; KAK3140413.1; [*Eleusine coracana*]	NA	NP
D–amino–acid transaminase; XP_006643973.2; [*Oryza brachyantha*]	NA	NP
Unnamed protein product; CAN8538735.1; [*Melica nutans*]	NA	NP
Unnamed protein product; CAN6330934.1; [*Urochloa humidicola*]	NA	NP
Unnamed protein product; CAN6340087.1; [*Urochloa humidicola*]	NA	NP
D–amino–acid transaminase; XP_062180852.1; [*Phragmites australis*]	NA	NP
D–amino–acid transaminase; XP_040376077.1; [*Oryza brachyantha*]	NA	NP
Hypothetical protein ZWY2020_033479; KAI5006236.1; [*Hordeum vulgare*]	NA	NP
D–amino–acid transaminase; XP_020164132.1; [*Aegilops tauschii*]	Aminodeoxychorismate lyase	–
D–amino–acid transaminase; XP_037405065.1; [*Triticum dicoccoides*]	NA	NP
Hypothetical protein E2562_020951; KAF0917577.1; [*Oryza meyeriana*]	Aminodeoxychorismate lyase	–
Hypothetical protein ACP70R_030771; KAL6627045.1; [*Stipagrostis hirtigluma*]	NA	NP
Unnamed protein product; CAN6353282.1; [*Urochloa humidicola*]	NA	NP
D–amino–acid transaminase; XP_044972325.1; [*Hordeum vulgare*]	Aminodeoxychorismate lyase	–
Unnamed protein product; CAN6326224.1; [*Urochloa humidicola*]	NA	NP
D–amino–acid transaminase; XP_044348217.1; [*Triticum aestivum*]	NA	NP
Hypothetical protein GUJ93_ZPchr0001g29627; KAG8052481.1; [*Zizania palustris*]	Uncharacterized protein	–
D–amino–acid transaminase; XP_020098053.1; [*Ananas comosus*]	Aminodeoxychorismate lyase	–
D–amino–acid transaminase; XP_037412366.1; [*Triticum dicoccoides*]	NA	NP
D–amino–acid transaminase; XP_010230671.1; [*Brachypodium distachyon*]	NA	NP
Hypothetical protein CFC21_040827; KAF7028988.1; [*Triticum aestivum*]	Aminodeoxychorismate lyase	–
D–amino–acid transaminase; XP_072998702.1; [*Typha latifolia*]	NA	NP
D–amino–acid transaminase; XP_062188721.1; [*Phragmites australis*]	NA	NP
D–amino–acid transaminase; XP_015621951.1; [*Oryza sativa*]	NA	NP
D–amino–acid transaminase; XP_072965437.1; [*Typha angustifolia*]	NA	NP
Hypothetical protein ACQ4PT_051529; KAM0852758.1; [*Festuca glaucescens*]	NA	NP
Hypothetical protein ACQ4PT_058457; KAM0842281.1; [*Festuca glaucescens*]	NA	NP
D–amino–acid transaminase; XP_010916006.1; [*Elaeis guineensis*]	Aminodeoxychorismate lyase	–
D–amino–acid transaminase; XP_034593875.1; [*Setaria viridis*]	NA	NP
D–amino–acid transaminase; XP_017697165.2; [*Phoenix dactylifera*]	Aminodeoxychorismate lyase	–
D–amino–acid transaminase; XP_026658547.2; [*Phoenix dactylifera*]	Aminodeoxychorismate lyase	–
D–amino–acid transaminase; XP_004967351.1; [*Setaria italica*]	NA	NP
Unnamed protein product; CAM0880605.1; [*Alopecurus aequalis*]	NA	NP
D–amino–acid transaminase; XP_072965436.1; [*Typha angustifolia*]	NA	NP
Hypothetical protein LUZ63_016396; KAJ1685006.1; [*Rhynchospora breviuscula*]	Aminodeoxychorismate lyase	–
Hypothetical protein VPH35_051201; XBI26505.1; [*Triticum aestivum*]	NA	NP
D–amino–acid transaminase; WOL06983.1; [*Canna indica*]	Aminodeoxychorismate lyase	–
Branched–chain–amino–acid aminotransferase–like protein 3; EMS47627.1; [*Triticum urartu*]	Aminodeoxychorismate lyase	–
Hypothetical protein GQ55_5G542800; PUZ58880.1; [*Panicum hallii*]	Aminodeoxychorismate lyase	–
Unnamed protein product; VAH59452.1; [*Triticum turgidum*]	Aminodeoxychorismate lyase	–
Unnamed protein product; CAL9118503.1; [*Musa acuminata*]	NA	NP
D–amino–acid transaminase; XP_009419654.2; [*Musa acuminata AAA Group*]	NA	NP
Unnamed protein product; CAL9769247.1; [*Musa acuminata*]	NA	NP
D–amino–acid transaminase; XP_025816942.1; [*Panicum hallii*]	NA	NP
D–amino–acid transaminase; XP_010260308.1; [*Nelumbo nucifera*]	D–amino–acid transaminase	–
D–amino–acid transaminase; RLM91444.1; [*Panicum miliaceum*]	Aminodeoxychorismate lyase	–
D–alanine aminotransferase; KAJ4765530.1; [*Rhynchospora pubera*]	Aminodeoxychorismate lyase	–
Unnamed protein product; CAL9057491.1; [*Musa banksii*]	NA	NP
D–alanine aminotransferase; KAJ4752619.1; [*Rhynchospora pubera*]	Aminodeoxychorismate lyase	–
D–amino–acid transaminase; XP_051224002.1; [*Lolium perenne*]	NA	NP
Unnamed protein product; VAH75043.1; [*Triticum turgidum*]	Aminodeoxychorismate lyase	–
D–alanine aminotransferase; KAJ4794410.1; [*Rhynchospora pubera*]	Aminodeoxychorismate lyase	–
D–amino–acid transaminase; XP_020249653.1; [*Asparagus officinalis*]	NA	NP
Hypothetical protein PVAP13_3KG076700; KAG2623703.1; [*Panicum virgatum*]	Uncharacterized protein	–
Hypothetical protein ACP4OV_020875; KAL6850248.1; [*Aristida adscensionis*]	NA	NP
D–amino–acid transaminase; XP_065035121.1; [*Musa acuminata AAA Group*]	NA	NP
Unnamed protein product; CAL9176660.1; [*Musa hybrid cultivar*]	NA	NP
D–amino–acid transaminase; XP_058095808.1; [*Magnolia sinica*]	NA	NP
D–amino–acid transaminase; XP_039811154.1; [*Panicum virgatum*]	NA	NP
Hypothetical protein QJS10_CPB20g01869; KAK1286490.1; [*Acorus calamus*]	Aminodeoxychorismate lyase	–
D–amino–acid transaminase; KAJ6791951.1; [*Iris pallida*]	Aminodeoxychorismate lyase	–
Unnamed protein product; CAG1852284.1; [*Musa acuminata*]	Aminodeoxychorismate lyase	–
D–amino–acid transaminase; XP_042503184.1; [*Macadamia integrifolia*]	NA	NP
Unnamed protein product; CAN8525773.1; [*Melica nutans*]	NA	NP
D–amino–acid transaminase; XP_020576401.1; [*Phalaenopsis equestris*]	NA	NP
Aminotransferase class IV; URE42738.1; [*Musa troglodytarum*]	Aminodeoxychorismate lyase	–
Branched–chain–amino–acid aminotransferase–like protein 3; KAM0784054.1; [*Phalaenopsis equestris*]	NA	NP

^a^NCBI nr protein database (https://www.ncbi.nlm.nih.gov/) search results. Search criteria: E-value < 0.01, shared identity > 50%, alignment size and gap frequency ≤ 6. No limiting keywords were used.

^b^Accessed at UniProt database(https://www.uniprot.org/) using the ID number.

^c^Searched through the combination of the species name source of the protein and the protein name in PubMed(https://www.ncbi.nlm.nih.gov/pubmed/).

^d^Not available.

^e^Not adverse effects reported.

^f^Not found.

This bioinformatic screening did not identify alignments meeting the search criteria with known protein toxins, allergens, or polypeptides associated with reported adverse human or animal health effects.^12^ The matched proteins were annotated mainly as D-amino-acid transaminases, aminotransferases, or aminodeoxychorismate lyases, or as hypothetical or uncharacterized proteins (Table 1). In summary, no matches to known toxins were identified, and the matches were to plant proteins, several of them from widely consumed species. Thus, these *in silico* findings add to the weight of evidence of safety of the OsDIAT protein.^11^

#### 3.5.2. Bioinformatics Prediction of Potential Allergenicity

To evaluate the potential allergenicity of the OsDIAT protein, bioinformatic analyses were conducted using three allergen databases. As detailed in Table 2, searches utilizing both the full-length OsDIAT sequence and the 80-aa sliding window methodology yielded no significant alignments with any known allergens across the evaluated databases. Notably, no sequence segment exceeded the 35% identity threshold within any 80-aa window, which is the criterion used to identify potential IgE cross-reactivity.^8^

**Table 2.** Summary of the allergenicity assessment of the rice (*Oryza sativa*) OsDIAT protein using various bioinformatics databases.

Database	Full length	80aa	6aa	7aa	8aa
AllergenOnline	0	0	0	0	0
ADFS	0	0	0	0	0
Allermatch^TM^	0	0	19	0	0

Furthermore, in the exact short contiguous amino acid search, no matches of 8 identical contiguous amino acids (8-mer) were identified. Although the 6-aa contiguous search detected 19 identical matches in the Allermatch™ database, each of these was an isolated single 6-aa match to an individual allergen (1 of 335 windows analyzed, i.e., 0.30%; Table 3), for example to allergens from the American cockroach (*Periplaneta americana*, Per a 18.01) and peanuts (*Arachis hypogaea*, Ara h 8.0101). Short 6-aa matches are statistically likely to occur by chance and are considered poor predictors of linear IgE-binding epitopes; this criterion is therefore known to generate false-positive results.^32,33^

**Table 3.** Results of the short contiguous 6-amino acid match search for OsDIAT against known allergens in the Allermatch^TM^ database.

Allergen name	Accession number	% Exact match	*Species*
Per a 18.01	UZC36341	0.30	*Periplaneta americana*
Fus c 1	Q8TFM9	0.30	*Fusarium culmorum*
Cla h 5	P42039	0.30	*Davidiella tassiana*
Alt a 5	P42037	0.30	*Alternaria alternata*
Cul n 1	Q9BMP6	0.30	*Culicoides nubeculosus*
Ara h 8.0101	Q6VT83	0.30	*Arachis hypogaea*
Zea m 25.0101	Q4W1F7	0.30	*Zea mays*
Vesp a 1	P0DMB5	0.30	*Vespa affinis*
Vesp a 1	P0DMB4	0.30	*Vespa affinis*
Alt a 3	P78983	0.30	*Alternaria alternata*
Sec c 5.0101	F4MJM3	0.30	*Secale cereale*
Hev b 9	Q9LEI9	0.30	*Hevea brasiliensis*
Hev b 9	Q9LEJ0	0.30	*Hevea brasiliensis*
Cyn d 4	Q5QJ60	0.30	*Cynodon dactylon*
Asp f 7	O42799	0.30	*Aspergillus fumigatus*
Asp f 4	O60024	0.30	*Aspergillus fumigatus*
Per a 11.0101	AKH04310	0.30	*Periplaneta americana*
No Allergen Name	A0A9E7VBU4	0.30	*Periplaneta americana*
Per a 11.0101	A0A0F7J1J1	0.30	*Periplaneta americana*

*The value 0.30 indicates the percentage of exact 6-amino acid word matches (1 exact match out of 335 analyzed windows, 1/335 × 100 ≈ 0.30%), as computed by the AllerMatch^TM^ system.

In conclusion, the *in silico* allergenicity assessment suggests that OsDIAT lacks significant sequence homology or structurally relevant immunogenic motifs shared with known allergens. These findings add to the weight of evidence that the OsDIAT protein has a low potential to act as a primary food allergen or to elicit cross-reactive allergic responses, which is consistent with its HOSU. However, these analyses assess only sequence similarity to known allergens and do not by themselves address the potential for *de novo* sensitization.

#### 3.5.3. Functional Domain Analysis and Literature-Based Hazard Identification

To further characterize the biochemical function of OsDIAT and to identify whether its sequence contains features associated with toxicity, structural and functional domain analyses were conducted using the InterPro database. Bioinformatic characterization indicated that OsDIAT is a canonical member of the pyridoxal phosphate (PLP)-dependent transferase superfamily (InterPro accession: IPR015424). Specifically, the protein sequence harbored a highly conserved D-amino acid aminotransferase-like domain (IPR001544), including a signature D-amino acid aminotransferase active site (IPR018151) and a PLP-binding pocket. These structural features are consistent with a typical endogenous metabolic enzyme that utilizes PLP as a cofactor to catalyze the reversible transamination of amino acids, a fundamental and ubiquitous metabolic process in plants. This domain analysis did not identify any structural motifs, virulence factors, or functional domains associated with known toxins, venoms, or anti-nutritional proteins.^12^

Complementing the *in silico* structural analysis, a literature-based hazard identification was performed using the PubMed database to screen for any previously reported adverse effects or safety concerns associated with the mode of action of the protein.^9^ The search strategy employed specific Boolean keyword combinations: (a) “OsDIAT” AND “mode of action”; (b) “OsDIAT” AND “specificity”; (c) “OsDIAT” AND (“inhibitor” OR “toxicity”); and (d) “mode of action” AND “D-amino-acid transaminase.”

The literature retrieval for combinations (a), (b), and (d) yielded no toxicologically relevant scientific publications, indicating a lack of reported safety concerns regarding the physiological mechanisms of the enzyme. Although the search for combination (c) returned several hits, review of the abstracts and full texts indicated that these studies were unrelated to the plant-derived OsDIAT protein and instead focused on general enzymatic inhibitors or toxicological phenomena in phylogenetically unrelated organisms. In conclusion, the functional domain architecture and the literature search are consistent with OsDIAT being a conventional metabolic enzyme. The absence of identified toxic domains and of literature-based hazard reports adds to the weight of evidence supporting the HOSU of the OsDIAT protein.^7^

#### 3.5.4. Prediction of Potential N-Glycosylation Sites

*In silico* prediction of potential N-glycosylation sites provides information relevant to the allergenicity assessment of plant proteins. Plant-specific N-glycans (e.g., α-1,3-fucose and β-1,2-xylose) can act as cross-reactive carbohydrate determinants (CCDs), which may elicit IgE-binding and result in false-positive allergic cross-reactivity during *in vitro* testing.^34^ The potential for post-translational N-glycosylation of the OsDIAT protein was evaluated using NetNGlyc-1.0. The bioinformatics algorithm identified two putative consensus motifs (Asn-Xaa-Ser/Thr) at amino acid positions 27 and 121, with prediction scores above the default threshold of 0.5. Because rOsDIAT was produced in *E. coli*, which does not perform plant-type N-glycosylation, the recombinant protein is not expected to be glycosylated, and the glycosylation status of the OsDIAT protein in plants cannot be assessed from the present data.

However, the physiological relevance of these predicted motifs must be contextualized based on the subcellular localization of the target protein. N-Glycosylation is a highly compartmentalized co- and post-translational modification that occurs exclusively within the lumen of the endoplasmic reticulum (ER) and Golgi apparatus.^35^ OsDIAT is an aminotransferase involved in primary amino acid metabolism. SignalP 6.0 did not predict an N-terminal signal peptide, and DeepTMHMM did not predict a transmembrane domain, in the OsDIAT sequence, suggesting that OsDIAT is not targeted to the secretory pathway (ER-Golgi); its subcellular localization was not experimentally verified in this study.

If OsDIAT does not enter the secretory pathway, it would be spatially separated from the ER-Golgi glycosylation machinery, and the predicted *in silico* N-glycosylation sites at positions 27 and 121 would be unlikely to be glycosylated *in vivo*. This suggests that OsDIAT may not be glycosylated in its native state. N-Glycosylation occurs mainly in the secretory pathway, and proteins that do not enter this pathway are generally not N-glycosylated.^35,36^ If confirmed experimentally for the plant-expressed protein, this would indicate a low likelihood of glycan-mediated cross-reactivity; this has not been verified.

### 3.6. Limitations of the Study

The present study has several limitations that should be considered when interpreting the results. (1) The digestion and heat-stability experiments were performed with the recombinant protein produced in *E. coli*; equivalence (e.g., molecular weight, immunoreactivity, enzymatic activity, and glycosylation status) between the recombinant protein and the plant-expressed protein has not been demonstrated, and the data therefore cannot be associated with any genetically modified plant. (2) The recombinant protein was purified under denaturing conditions and urea was subsequently removed by dialysis; recovery of the native conformation and enzymatic activity was not verified. (3) Heat stability was evaluated at a single temperature (95 °C), and enzymatic activity under heat treatment was not measured. (4) Digestion in SGF and SIF was evaluated in separate time-course assays; sequential SGF–SIF digestion was not performed, and fragments that do not retain the C-terminal His-tag, as well as small peptides, were not detected. (5) The *in silico* analyses are predictive and are based on sequence similarity only, and the literature search did not identify protein-specific safety studies; no experimental assessment of toxicity or allergenicity (e.g., IgE binding) was performed, and these data represent only part of the weight of evidence. Once equivalence with the plant-expressed protein has been demonstrated, the present data may contribute to the weight of evidence for the safety assessment of future GM events expressing this protein.

## 4. Conclusions

In the present study, a preliminary characterization of the recombinant OsDIAT protein was conducted, together with *in silico* and literature-based analyses, to contribute to the weight of evidence supporting the HOSU of the OsDIAT protein.^8,10^ *Oryza sativa*, the source organism, has a long history of human consumption and is a global staple food. In the assays performed, the intact recombinant OsDIAT protein was no longer detectable within 1 min in SGF, whereas it remained detectable in SIF for 60 min in a separate time-course assay, and it was no longer detectable from 60 min of heating at 95 °C (the only temperature tested); enzymatic activity was not measured.^11^ The *in silico* analyses identified no significant sequence similarity to known toxins or allergens,^12^ and the predicted N-glycosylation sites are considered unlikely to be modified, given that no signal peptide or transmembrane domain was predicted, although this has not been verified experimentally.

In conclusion, the integration of *in vitro* digestion, thermal stability, and bioinformatics data adds to the weight of evidence supporting the HOSU of the OsDIAT protein, but it does not by itself constitute a safety assessment. The data presented in this study characterize only the microbially produced recombinant protein and have no bearing on any genetically modified plant until equivalence between the plant-expressed protein and the recombinant protein has been demonstrated. Further studies, including demonstration of such equivalence, evaluation of enzymatic activity under heat treatment across a range of temperatures, and sequential SGF–SIF digestion, are recommended. Once equivalence has been shown, the present data may contribute to the weight of evidence for the safety assessment of future GM events expressing this protein.

## Supplementary Material

Supplementary Information.docx
