# Supplementary material for: Establishing the history of safe use of the OsDIAT protein: preliminary characterization of the recombinant protein and in silico analyses
Source: GM Crops Food. 2026 Nov 4;17(1):2753265. doi: 10.1080/21645698.2026.2753265 (PMC13757565; doi:10.1080/21645698.2026.2753265)
Supplement: Supplementary file 1 [file KGMC_A_2753265_SM6770.docx]

**Supplementary Information**

**Title: Comprehensive food safety assessment of the recombinant OsDIAT protein for the development of climate-resilient transgenic soybean**

**Table S1.** Primers used for cloning.

| Locus ID | Primer name | Primer sequence (5`→3`) |
| --- | --- | --- |
| Os05g0244700 | DIAT_Forward | 5`-ATGCGGCCGCATGCAAGGGGAACACCATGA-3` |
|  | DIAT_Reverse | 5`-ATCTCGAGCTAGCTCGCTTGATCATAAG-3` |

**Figure S1.**

**Figure S1. Schematic map of the recombinant expression vector pET-26b(+)-DIAT.**

The full-length coding sequence of the *OsDIAT* gene (Locus ID: Os05g0244700) was cloned into the E. coli expression vector pET-26b(+) between the restriction sites. The construct features an N-terminal histidine tag (6xHis) for affinity purification under the control of the T7 promoter and lac operator. Key genetic elements including the kanamycin resistance marker (*KanR*), T7 terminator, ribosome binding site (RBS), and origin of replication (*ori*) are indicated with their respective relative positions (total length: 6,248 bp).
